# Interplay between Cellular and Non-Cellular Components of the Tumour Microenvironment in Hepatocellular Carcinoma

**DOI:** 10.3390/cancers13215586

**Published:** 2021-11-08

**Authors:** Tamás Sükei, Elena Palma, Luca Urbani

**Affiliations:** 1 The Roger Williams Institute of Hepatology, Foundation for Liver Research, London SE5 9NT, UK; tamas.1.sukei@kcl.ac.uk (T.S.); e.palma@researchinliver.org.uk (E.P.); 2 Faculty of Life Sciences and Medicine, King’s College London, London WC2R 2LS, UK

**Keywords:** extracellular matrix, liver cancer, tumour microenvironment, bioengineering, 3D models

## Abstract

**Simple Summary:**

The tumour microenvironment comprise cellular and non-cellular components and is a dedicive factor in determining anti-cancer treatment efficiency. The extracellular matrix is profoundly remodelled in cancer, with direct effects on cancer cell growth and local immunity. In this review, we outline how the matrix is altered in liver cancer and the importance of including the matrix and other features of the tumour microenvironment in disease models.

**Abstract:**

Hepatocellular carcinoma (HCC) is one of the most common and lethal cancers worldwide. Currently, treatments available for advanced HCC provide dismal chances of survival, thus there is an urgent need to develop more effective therapeutic strategies. While much of the focus of recent decades has been on targeting malignant cells, promising results have emerged from targeting the tumour microenvironment (TME). The extracellular matrix (ECM) is the main non-cellular component of the TME and it profoundly changes during tumorigenesis to promote the growth and survival of malignant cells. Despite this, many in vitro models for drug testing fail to consider the TME leading to a high failure rate in clinical trials. Here, we present an overview of the function and properties of the ECM in the liver and how these change during malignant transformation. We also discuss the relationship between immune cells and ECM in the TME in HCC. Lastly, we present advanced, 3D culture techniques of cancer modelling and argue that the incorporation of TME components into these is essential to better recapitulate the complex interactions within the TME.

## 1. Introduction

Liver cancer is the sixth most common form of cancer in incidence worldwide across both sexes and all ages and in 2020 there were 900,000 cases worldwide. It is the third in cancer-related deaths, claiming more than 800,000 lives globally in 2020 [1]. With incidence on the rise worldwide, it is estimated that by 2030 over 1 million people will die from liver cancer [2]. The most common form of primary liver cancer is hepatocellular carcinoma (HCC) that represents 90% of cases [3]. The survival rate for HCC is poor, with a 5-year rate standing at 18% [4]. Moreover, 90% of HCC cases develop on the back of persistent liver inflammation which could result in aberrant chromosomal changes and can lead to the malignant transformation of hepatocytes [5,6]. The most important risk factor for HCC is cirrhosis as one in three cirrhotic patients will develop HCC during the course of their lives [7]. Other prevalent risk factors for HCC are hepatitis B (HBV) or hepatitis (HCV) infections, excessive alcohol consumption, obesity-related or diabetes non-alcoholic steatohepatitis (NASH), aflatoxin B1, and these risk factors vary by geographical region [3].

HCC is a molecularly highly heterogeneous malignancy and this aspect is present at three levels: interpatient heterogeneity, intertumoural heterogeneity (variability within the tumour nodules of the same patient) and intratumoural heterogeneity (variability between different regions of the same tumour nodule) [8]. This high heterogeneity coupled with the suppressive tumour microenvironment [9] makes creating a universally effective treatment challenging. Currently, treatment is determined by scoring on the Barcelona Clinic Liver Cancer algorithm. Stage 0 and A patients are eligible for surgical resection, however, 70% of patients undergoing resection will have a recurrence within 5 years [10]. Patients with stages B (intermediate) and C (advanced) HCC have systemic therapies available that mainly consists of various multi-kinase inhibitors (e.g., sorafenib, lenvatinib) [11]. Immune checkpoint inhibitor (ICI) monotherapy of cytotoxic lymphocyte antigen-4 (CTLA-4) or programmed cell death (PD-1)/programmed cell death ligand-1 (PD-L1) showed promising results in early clinical trials [12,13,14,15] which resulted in the FDA granting approval to pembrolizumab and nivolumab, now recommended as 3rd line of treatment [11]. However, median overall survival (OS) and objective response rates (ORR) were 1 year and 15% for PD-1/PD-L1 blockade in patients previously treated with sorafenib [12,13], whereas for CTLA-4 blockade the median time to progression was 6.48 months and the ORR was 17.6% [15]. In addition, further randomised trials for anti-PD-1 monotherapy for HCC did not show statistically increased OS either as a first-line treatment (against sorafenib) [16] or as a second-line treatment (against placebo) [17]. However, combination therapy of ICI and other agents have shown promising results. In a global phase 3 clinical trial, atezolizumab (PD-1 inhibitor) was administered alongside bevacizumab (VEGF inhibitor) to patients with unresectable HCC and the combination therapy had better OS at 12 months (67.2% vs. 54.6%) and median progression-free survival (6.8 vs. 4.3 months) than patients given sorafenib only [18]. Other combinations include pembrolizumab (PD-1 inhibitor) and lenvatinib (multi kinase inhibitor) [19], atezolizumab and cabozantinib (multi-kinase inhibitor) [20] or the combination of different ICIs such as durvalumab and tremelimumab [21] and nivolumab and ipilimumab [22].

## 2. The Extracellular Matrix in the Tumour Microenvironment

The positive effect of immunotherapy, when combined with kinase inhibitors, highlights the importance of the microenvironment in HCC. The tumour microenvironment (TME) in solid tumours is made up of the tumour cells and tumour-associated stroma [23]. The tumour stroma comprises cellular components such as blood and lymphatic vessels, cancer-associated fibroblasts (CAFs) and immune cells, as well as non-cellular components such as the extracellular matrix (ECM) [23]. While much valuable information has been extracted from 2D cell culture models of HCC, they fail to account for various important environmental factors that can affect HCC progression and therapy. One such factor is the change in composition and mechanical properties of the ECM.

### 2.1. The Liver ECM in Health

The ECM is a complex assembly of core proteins such as collagens, fibronectin and glycoproteins (such as heparan sulphate proteoglycans, chondroitin sulphate proteoglycans) and other associated molecules such as growth factors, cell adhesion molecules and cytokines [24]. The ECM is an important constituent of healthy tissues that provides structural support and is involved in various physiological processes including tissue homeostasis [25], cell migration [26] signalling [27] and differentiation [28]. These biochemical cues provided by the ECM are tissue specific, as they vary in degree and characteristics depending on the specific tissue type and function [29]. In the healthy liver, ECM components are synthesised by a variety of cells. Hepatic stellate cells (HSCs), sinusoidal endothelial cells and hepatocytes produce type IV collagen and several glycoproteins and proteoglycans. In addition, fibroblasts, vascular endothelial cells, biliary epithelial cells in the portal tracts and myofibroblast surrounding the central veins also synthesise ECM constituents [30]. The healthy human liver is comprised over 150 ECM proteins [31] and both the parenchymal and the stromal cells can secrete factors that can regulate ECM density and can modulate the immune system. Matrix metalloproteinases (MMPs) are calcium-dependent zinc-containing endoproteinases that degrade ECM constituents and are secreted by different hepatic cell types including HSCs, Kupffer cells (KCs), bile ductular epithelial cells and hepatocytes [32]. There is a low level of MMP expression in healthy individuals that helps maintain tissue homeostasis and their activity is regulated by tissue inhibitor of matrix metalloproteinases (TIMPs) [33]. Other metalloproteinases including disintegrins and metalloproteinases (ADAMs) or disintegrin and metalloproteinases with thrombospondin motifs (ADAMTs), are also important in degrading the ECM and contributing to its homeostasis [34].

### 2.2. A Remodelled ECM in Liver Cancer

The typical remodelling of the ECM that occurs in cancer development and growth serves as a supportive environment for tumorigenesis, the maintenance of cancerous tissue and metastasis. ECM remodelling is driven by various processes in cancer [35], summarized in Table 1. Commonly, there is an accumulation of ECM components such as collagens in the tumour stroma [36]. Ma et al. demonstrated that COL1A1 is highly expressed in HCC and can be used as a putative biomarker for HCC carcinogenesis and metastasis [37]. In addition, it has been shown that HSCs trigger the epithelial to mesenchymal transition (EMT) of hepatocellular carcinoma cells via the secretion of type I collagen [38]. Overtly abundant collagen can also increase cancer cell survival. By binding to integrins, it increases focal adhesions, initiates PI3K signalling and promotes the growth of tumour cells [39].

There is elevated deposition of other stromal components such as hyaluronic acid (HA) and its receptor CD44 that together increase growth factor signalling [40]. Other components such as heparan sulphate proteoglycans are also excessively produced [36] and due to their involvement in cancer signalling, proteoglycans have been proposed as potential biomarkers and therapeutic candidates in HCC [41]. The excessive secretion of ECM components increases matrix stiffness that further aids cancer progression. Higher matrix stiffness upregulates vascular endothelial growth factor (VEGF) in HCC cells, which increases tumour growth and invasiveness [42]. Furthermore, increased matrix stiffness in HCC upregulates osteopontin (OPN) expression [43] which is known to worsen HCC prognosis [44].

Post-translational modifications (PTMs) are also altered in the cancer milieu. ECM proteins undergo PTM after synthesis and the disruption of these processes can aid malignant transformation of the tissue [45]. Lysyl oxidase (LOX) family members cross-link collagen with elastin fibres and are thus critical regulators of ECM stiffness. LOX has been shown to be overexpressed in HCC and that this elevated expression correlates with invasiveness and poor survival [46,47].

ECM degradation is also perturbed in HCC. In normal, homeostatic tissue, the ECM is continuously degraded by proteases such as MMPs, ADAMs or ADAMTs [48]. However, in cancer tissues, there is elevated expression and activity of MMPs [48] and in HCC, various MMP levels are elevated [49]. MMP-2 and MMP-9 are prominent in HCC; MMP-2 is not typically found in the healthy liver but is expressed in HCC and is linked to increased invasiveness while MMP-9 promotes angiogenesis [49]. Importantly, MMPs not only contribute to tumour progression by opening up passageways for metastasis but also by creating new bioactive molecules termed matrikines [50]. These matrikines can promote tumour progression although some have anti-tumorigenic effects. For example, AG-9, an elastin fragment upregulates tumour growth in melanoma but A5G27, a laminin fragment reduced breast cancer cell proliferation [51]. MMP-12 expression has been associated with reduced tumour vascularisation in HCC [52,53]. This protective effect of MMPs has been shown in other cancers as well. MMP-8 expression was correlated with better overall survival in squamous cell carcinoma [54], while MMP-9 was shown to be protective against Lewis lung carcinoma [55]. 

Lastly, the increased stiffness of the ECM can affect ECM-integrin interactions inducing mechano-signalling and increasing cancer invasiveness [39]. Increased stiffness has been shown to induce upregulation of integrin-ß1 expression in HCC and increase invasiveness [56]. Matrix stiffness was also shown to be sufficient on its own to induce EMT in HCC and this could be reversed by integrin ß1 or α5 suppression [57]. High matrix stiffness also increased resistance to sorafenib in breast cancer through integrin ß1 and its downstream effector JNK [58]. Similar results were observed in HCC, where cells grown on a higher substrate displayed increased resistance to the chemotherapeutic agent cisplatin [59].

Significant producers of ECM material in cancer are CAFs, which are spindle-shaped cells that deposit and remodel the ECM [60,61]. CAFs are a poorly defined population of cells, due to their heterogeneity and the absence of specific markers that can delineate them [62]. They have a central role in desmoplasia by secreting various ECM components including collagen types I, III, IV, V, hyaluronan, fibronectins, laminins as well as secreting proteases such as MMPs and urokinase-type plasminogen activator (uPA) [62]. Their excessive secretion of ECM components results in the ECM becoming stiffer, shielding the tumour from the immune system and supporting tumour growth. ECM degradation by CAF secreted MMPs allows the liberation of various ECM bound factors, such as VEGF-A which promotes angiogenesis [62]. CAFs are also able to influence the immune response to tumours by secreting a plethora of growth factors, cytokines and chemokines such as transforming growth factor-beta (TGF-β), interleukin 6 (IL-6) or CCL2 [62]. By doing so in HCC they can induce the generation of myeloid-derived suppressor cells (MDSCs) [63] or suppress NK cell activation [64]. CAFs have several cellular sources in hepatic cancer. A common one is HSCs that are normally quiescent, vitamin A storing cells that line the perisinusoidal space [65]. However, they are commonly transformed into alpha-smooth muscle actin (αSMA) expressing myofibroblasts in the TME which are then localised around tumour sinusoids, fibrous septae and the tumour capsule [66]. HCC tumour cells can also undergo EMT and be transformed into CAFs often displaying aSMA or fibroblast activation protein (FAP) expression, and are also associated with high aggressiveness [67]. Other cell types could also be transformed into CAFs, these include mesenchymal stem cells (MSCs), liver sinusoidal endothelial cells (LSECs) and peritumoural tissue fibroblasts (PTFs) [67].

### 2.3. Cytokines Are Key Regulators of the Tumour Microenvironment

Since HCC development is heavily linked to chronic inflammation [68], it is important to consider the role of cytokines especially those with pro-inflammatory properties in hepatic tumorigenesis. Cytokines are small, intercellular signalling molecules that play a key role in orchestrating and resolving inflammation [69]. The dysregulation of their production is a central feature of the hepatic TME [68]. An important driver of inflammation is IL-6 whose levels are elevated in the serum of HCC patients [70]. IL-6 initiates JAK/STAT signalling, a pathway central to various physiological processes and it is a crucial player in steatohepatic HCC [70]. Another crucial cytokine in the HCC TME is TGF-β. TGF-β acts as a suppressor in the early stages of tumour development, however, in later stages, it becomes tumour promoting also driving EMT [71]. Moreover, TGF-β modulates the immune landscape within the TME: it skews T cells towards a Treg phenotype, and it also regulates other immune cells such as tumour associated macrophages (TAMs), MDSCs or NK [72]. Other cytokines such as VEGF and IL-10 are also important in the HCC TME. VEGF is a prominent angiogenic factor and its expression is elevated in HCC [73] contributing to its hypervascularisation. VEGF levels in the serum of HCC patients have been correlated with tumour invasiveness [74] and have also been shown to serve as a predictive factor for the success of therapy [75]. Serum levels of the anti-inflammatory cytokine IL-10 have also been found to negatively correlate with overall survival [76]. The role of cytokines in HCC development is extensively reviewed elsewhere [77].

### 2.4. The Effect of the Remodelled ECM on the Tumour Immune Microenvironment

An important component of the TME is represented by infiltrating lymphocytes, creating the so-called tumour immune microenvironment (TIME). Research suggests that there are two main types of TIME present in solid tumours. Infiltrated-excluded (I-E) TIMEs are generally populated with immune cells but mainly lack cytotoxic lymphocytes (CTL) in the tumour core [78]. CTLs are typically positioned around the tumour core likely stuck in the surrounding dense ECM. I-E TIMEs are considered to be immunologically ‘cold’ since CTLs in them have low expression of activation markers granzyme B and IFNG and they have poor CTL infiltration into the tumour core [78]. This TIME type is often found in epithelial carcinomas such as melanoma, pancreatic ductal adenocarcinoma (PDAC), colorectal cancer [78]. Infiltrated-inflamed (I-I) TIMEs are characterized by high infiltration of CTLs into the tumour core that express PD-1 and the TME expressing PD-L1. This TIME is considered to be immunologically ‘hot’ and tumours with this phenotype show better response to ICI [78]. A subtype of this phenotype is the so-called tertiary lymphoid structures TIME in which there are lymphoid aggregates with similar cellular composition to lymph nodes that are usually found in the invasive tumour margin and in the stroma. They contain a variety of lymphocytes comprising Tregs, B cells, DCs and naïve and activated conventional T cells [79]. This subtype of TIME is usually associated with a positive prognosis [78]. A meta-analysis of 23 studies on the effect of tumour infiltrating lymphocyte (TILs) on the prognosis in HCC patients concluded that high levels of CD8+ and CD3+ TILs improved OS and that high levels of CD8+, CD3+ and CD4+ TILs were linked to improved disease-free survival/recurrence-free survival. On the other hand, FoxP3+ TILs levels and FoxP3+/CD4+ and FoxP3+/CD8+ ratios negatively correlated with OS and disease-free survival/recurrence-free survival [80]. An analysis of 956 HCC patients found that almost 25% of them showed markers of inflammation associated with a good response to anti-PD-1 therapy. Within this group, two subclasses were identified: a so-called ‘active immune response’ class that was characterised by an enrichment of IFN signatures, an overexpression of adaptive immune response genes and better survival (i.e., I–I), and a so-called ‘exhausted immune response’ class that was characterised by tumour promoting signals such as activated stroma, T-cell exhaustion and immunosuppressive components especially TGF-β (i.e., I–E) [81]. Gao et al. investigated the tumour immune signature of 766 HCC patients from three publicly available cohorts and found that the TIME could be clustered into four distinct subclasses that can influence response to treatment [82]. A third of patients belonged to the “immune deserted” or cold tumour class that is characterised by general immune ignorance. However, 17% of patients belonged to the “immunogenic” or hot tumour class that demonstrated enrichment of both innate and adaptive immune responses and showed the highest response to sorafenib and pembrolizumab treatments [82]. Similar clustering of TIMEs has been observed in melanoma, bladder and gastric cancers as well [83]. These findings illustrate just how important the microenvironment is in directing the immune response against the tumour.

The ECM is known to harbour proteins with immunomodulatory properties [84]. A prominent ECM-bound molecule is TGF-β1, which is secreted as a homodimer attached to its latency-associated peptide (LAP) to prevent TGF-β1 from binding to its receptors. TGF-β1 is then anchored to the ECM by various latent TGF-β1 binding proteins (LTBPs) that share sequence and domain similarities with fibrillins. Latent TGF-β1 can then be liberated by proteolytic degradation or by mechanical force [85]. TGF-β is a pleiotropic cytokine and it plays important roles in liver fibrosis and carcinogenesis by inducing the transformation of HSCs into myofibroblasts and by inducing EMT in hepatocytes [86]. Moreover, TGF-β also has various effects on anti-tumour immunity. It directly inhibits the hallmark of cytotoxic T cell anti-tumour immunity by suppressing perforin, granzyme A, granzyme B, Fas ligand and interferon-gamma (IFN-γ) production [87]. In addition, TGF-β is an important growth factor for the differentiation of CD4+ T-cells into regulatory T cells (Tregs) [88,89] that are important in dampening the fibrotic response.

The ECM includes proteins that have a variety of effects on the immune system and there is increasing evidence pointing to the role that ECM proteins play in immune exclusion. As a result of increased density, it plays a crucial role in T cell exclusion. In fresh, human lung ex vivo tumour slices, T-cells preferentially accumulated in the stroma with 5 times more T-cells there than in the tumour [90]. T cells were able to migrate better in looser collagen and fibronectin regions and collagenase treatment reversed the obstructing effects of the tumour stroma on T-cell migration [90]. Moreover, T-cells are known to migrate by reorganising their cytoskeleton that results in considerable cellular deformations allowing them to migrate through narrow spaces. However, when they are confronted by dense ECM they are unable to migrate through them and as a result, they migrate away towards looser ECM [91]. Treatments that target the components of the ECM could help: for example, collagenase treatment has been shown to increase T cells and tumour cells interactions [90]. Tenascin-c, a glycoprotein whose level increases in the tumour ECM, also helps trap T-cells in the stroma by binding to Toll-like receptor 4 (TLR-4) and inducing the upregulation of CXCL12 expression [92]. In a mouse pancreatic ductal adenocarcinoma (PDAC) model, antifibrotic treatment reduced hyaluronan concentration allowing a greater infiltration of immune cells into the tumour [93]. In pancreatic cancer, T cells accumulated in low collagen density areas and their ability to invade the tumour was inversely proportional to the density of the collagen matrix [94]. Moreover, a dense collagen network was able to abrogate chemokine-induced T-cell migration as well [94]. In a 3D model, high collagen density reduced T-cell proliferation, promoted CD4+ T cells over CD8+ T cells and reduced cytotoxic activity [95].

In addition to forming a physical barrier, ECM proteins can also directly modulate immunity. Collagen has been shown to lead to CD8+ T cell exhaustion through its binding to leukocyte associated immunoglobulin-like receptor 1 (LAIR-1) and to promote anti-PD-1/PD-L1 resistance [96]. OPN that is commonly abundant in the TME and peripheral blood of cancer patients has been shown to suppress CD8+ T cell activity in colon cancer and that such suppression can be overcome by OPN blockade [97].

In gingival squamous carcinoma (GSCC), high galectin-1 expression in tumour tissue correlated with decreased infiltration and increased apoptosis of CD8+ T-cells [98]. In head and neck cancer, high galectin-1 expression upregulated PD-L1 expression on the tumour epithelium reducing T-cell infiltration into the tumour [99]. In PDAC, galectin-9 binding of macrophage-expressed Dectin-1 has induced their pro-tumorigenic reprogramming and it caused the suppression of adaptive cancer immunity [100].

Treatments that target the components of the ECM could help: for example, collagenase treatment has been shown to increase T cells and tumour cells interactions [90]. A study has also shown that non-response to the anti-PD-L1 antibody atezolizumab in a cohort of metastatic urothelial cancer patients was correlated with the exclusion of CD8+ T cells from the tumour and their accumulation in the tumour stroma [101].

The dense ECM can also cause hypoxia, which can result in the upregulation of angiogenic factors such as VEGF. Among the plethora of immune-modulatory properties of this growth factor [102], VEGF is able to downregulate the expression and can also inhibit the clustering of intercellular adhesion molecule-1 (ICAM-1) and vascular cell adhesion molecule-1 (VCAM-1) [103,104]. These molecules are important in the extravasation of T cells as their expression and clustering on endothelial cells determines if T cells can extravasate into the tumour [105]. This effect of VEGF can be overcome by the use of VEGF inhibitors [106]. Dense ECM-induced hypoxia is also key to drug resistance and HCC is one of the most hypoxic tumour types [107]. The hypoxic environment causes the upregulation of hypoxia-inducible factors such as hypoxia-inducible factor 1 subunit alpha (HIF-1a) and HIF-2a that promote the resistance to sorafenib treatment [108]. Sorafenib’s antivascular effect ironically drives resistance to it by decreasing vascularisation, thus increasing hypoxia and promoting the selection of resistant cells [108].

## 3. Models to Study the Cellular and Non-Cellular Components of the TME

The role of the ECM in the TME is pivotal for drug development. Over half of all drugs in Phase II and Phase III clinical trials are unsuccessful [109] and currently the most used way of testing drug candidates is 2D cell culturing [110].

Two-dimensional (2D) cell cultures include primary cells and immortalised cell lines and while primary cells are valuable because they retain donor-specific features, their use is limited by slow growth and a short lifetime. Immortalised cell lines on the other hand can multiply indefinitely, but this makes them less representative of the original tumour. Moreover, the longer these cells are passaged the higher the chances of genetical and phenotypical changes that can affect results [111]. While 2D culturing techniques serve as a cost-effective way of testing compounds, they cannot reconstitute the heterogeneity of the tumour or the complexity of the extracellular environment [111] and this can hinder successful drug development. Three-dimensional models confer various advantages over conventional 2D culturing methods and these are key factors to explain why drugs show a different response in a 3D culture environment. Two-dimensional cultures lack the same morphological organisation as 3D cultures [112]. The spatial organisation of cell surface receptors is different to cells cultured in 3D and this could affect drug binding efficiency [113]. Cancer cells cultured in 3D better recapitulate the in vivo environment where cells are in various different stages of their life cycle [113]. It has been shown that tumour cells de-differentiate in 2D culture whereas in 3D they resemble closer in vivo morphologies [114,115]; cancer cells in spheroid cultures display increased angiogenic factors compared to cells in 2D cultures [116] and that cells cultured in 2D have lower IC50 values for drug treatment than cells cultured in a 3D environment [117]. Recently, a study analysed the metabolome of mouse inner medullary collecting duct cell line (mIMCD3) grown as spheroids and compared it with the metabolome of freshly isolated cells from the mouse distal ducts [118]. They found that the metabolome of cells grown in spheroids was analogous to freshly isolated cells and that cells grown in a 2D environment had a vastly differing metabolome.

Signalling is also different in a 3D environment. Head and neck cancer cells displayed upregulation of CDH1, Nanog and Sox2 when cultured in spheroids compared to when cultured in 2D [119]. Colon cancer cells have also shown a downregulation of AKT, mTOR and S6K signalling in spheroid culture compared to 2D culturing [120].

Since 3D cultures are more representative of the in vivo environment, they represent better systems to model cell–ECM and cell–cell interactions and their use is becoming more frequent. Currently, the most developed models of cancer that introduce the presence of a TME are represented by organoids, spheroids, organ-on-chips, precision-cut tissue slices and bioengineered tumour models, reported in Table 2. 

### 3.1. Organoids

An organoid is a collection of organ-specific cell types that is derived from stem cells or organ progenitors and is able to self-organise through cell sorting and spatially restricted lineage commitment [121]. The criteria for organoids are that it is able to display organ-specific functions and that cells in them are grouped together and organised in a similar fashion to the organ in vivo [121]. Organoid cultures have successfully been established for various tissue types including lung, ovarian, uterine, colorectal, bladder, liver, breast and biliary tract cancers [111]. The first functioning, healthy, human liver organoid was established in the Clevers lab [122] and the same group subsequently established human primary liver cancer organoids as well [123]. Huch and colleagues demonstrated that long-term organoid culture can be set up from primary liver cancer samples and that these organoids faithfully represent the phenotypical and genetic characteristics of the in vivo tumour they were derived from [123]. Liver organoids have proven to be a good predictive platform for drug efficiency. In a screening of 29 anticancer compounds on primary liver cancer organoids, an ERK 1/2 inhibitor has demonstrated efficacy. This effect was also recapitulated in vivo when these organoids were engrafted in immunodeficient mice [123]. A limiting factor of current organoid technology is the difficulty to support the maintenance of patient-derived stromal and immune cells along with tumour cells in culture [124]. An organoid culture derived from the mouse small intestinal crypts was successfully co-cultured with murine intestinal epithelial lymphocytes (IELs). These IELs could be expanded when IL-2, IL-7 and IL-15 were added and were found both inside and outside of the organoids [125]. CD45+ cells have successfully been maintained in human air-liquid interface tumour organoid cultures for up to 8 days, however, CD3+ cells showed a noticeable decrease during the same culture length [124]. The organoid technology could be particularly valuable in drug screening. Patient-derived organoids from colorectal liver metastasis (CRLM) patients have been shown to be predictive of response to chemotherapy [126,127] and can be utilised for drug response profiling [128]. The co-culture of CAFs with primary liver cancer organoids protected tumours from various clinically used anticancer agents [129]. Despite the potential presence of various cell types, the 3D organisation and the patient specificity are certainly an advantage in drug development [111], organoids have several drawbacks. These include high inter-patient variability, high cost, difficulty in achieving maturation and the lack of vascularisation [111,121].

### 3.2. Spheroids

Spheroids are also a popular 3D culturing method. They are formed by self-assembled, cell aggregates that have no attachment to a flat surface [130]. Spheroids lack a matrix constituent [111]. Their greatest advantage is that they can be created from immortalised cell lines as well as from primary cells thus they can also be patient specific. They are highly reproducible as well and can be cultured with the use of simple protocols [111]. Numerous experiments utilising spheroid culturing have shed light on the importance of the microenvironment. It has been demonstrated that HCC spheroids grew larger when co-cultured with activated HSCs and that they had a smaller necrotic core than when grown in a spheroid monoculture [131]. The addition of human umbilical vein endothelial cells (HUVECs) to Huh7-formed spheroids promoted their proliferation and increased the gene expression of HCC-related genes and cancer stem cell markers. More importantly, however, it was also demonstrated that this in vitro setup was also able to tolerate a higher dose of anti-cancer drugs than cells cultured in a monolayer [132]. Similar effects were observed when the HSC cell line LX2 was added to HCC spheroids formed by both primary cells and cell lines. Co-culture with HSCs not only increased spheroid compactness and resistance to chemotherapeutic agents and kinase inhibitors, but activated HSCs promoted HCC migration by upregulating MMP-9 as well [133]. Moreover, spheroids have been constructed from cell lines derived from HBV+ patients and co-cultured with a variety of stromal cells including stellate cells, HUVECs and fibroblasts. Spheroids co-cultured with stromal cells showed an altered response to anti-cancer drugs compared to spheroids cultured on their own or cells cultured in a monolayer [134]. However, spheroids have poor structural organisation, they form a necrotic core as a result of poor oxygen and nutrients supply, are variable in sizes and have limited viability due to the absence of progenitor cells [111].

### 3.3. Organ-on-Chip

Another option for 3D culture is organ-on-chips, which are microfluidic devices with individual parenchymal and vascular compartments containing live cells that model the tissue–tissue interfaces, the multicellular architecture, the physical microenvironment of the organ in vivo while having dynamic vascular perfusion [135]. Organ-on-chip technology provides a controlled environment and the ability for co-culture. While it has allowed for the functional recreation of several organs [136,137,138], it has been difficult to integrate immune organs largely due to their highly complex nature [139]. This means that studying certain aspects such as T-cell priming in the lymph nodes is not yet currently possible. Moreover, this technology has several hurdles including low throughput, technically challenging use, lack of technical robustness and difficulty of maintaining cell viability over an extended period of time [140]. Nonetheless, organ-on-chip technology has yielded valuable insights into how tumours interact with immune cells. Ayuso et al. have shown that MCF-7 tumours drive NK cells exhaustion and that this could be rescued by ICI treatment [141]. Aref et al. used both human and mouse-derived spheroids from various solid cancers in a microfluidic system [142]. They found that the spheroids retained autologous tumour infiltrating CD8+ T cells and that combination treatment with anti-PD-1 and anti-CTLA-4 antibodies promoted their expansion and increased tumour killing. Businaro et al. showed that splenocytes from wild-type mice showed a higher ability to migrate and to extravasate towards B16 melanoma cells than splenocytes from interferon regulatory factor-8 (IRF-8) deficient mice [143]. In addition, organ-on-chip technology is frequently used to model other aspects of cancer biology such as neovascularisation, modelling response to chemotherapy or tracking cancer cell migration and extravasation (reviewed in [140]).

### 3.4. Precision Cut Tissue Slices

Precision cut tissue slices (PCTS), which include liver (PCLS) and tumour slices, represent a valuable addition to the aforementioned 3D models as they allow the investigation of the interplay between the cellular and non-cellular components of the tissue in a nearly untouched physiological system. PCTS are fragments derived from fresh explants of liver or cancer. Tumour slices have been successfully obtained from multiple solid tumours, including pancreatic, renal, colon, breast [144,145,146,147] and have shown remarkable similarities with the clinicopathological parameters observed in patients [148,149]. PCTS can also be derived from primary and secondary liver cancers, including HCC specimens [150] and promising results indicate that the structural and metabolic signatures observed in vivo are retained within the PCTS [151,152].

The original tissue to produce PCTS is usually sourced from rodents or patients as surgical waste post resection of liver cancers and distal surrounding tumour-free tissue. The explants are then promptly processed in the laboratory to obtain slices nearly identical in size, using a specialised tissue microtome [153,154]. Liver or tumour slices can be maintained in culture for several days without significant loss in cell viability and functionality [155].

The preservation of cell-type heterogeneity is a key advantage of the PCTS model. Resident or infiltrating cell populations and parenchymal or stromal components are maintained in the PCTS, such as hepatocytes, hepatic stellate cells, fibroblasts, or tumour cells and tumour infiltrating lymphocytes. Furthermore, an intact ECM is retained, and so are the intra- and inter-cellular interactions allowing the use of this model to study the TME [156]. Notably, the presence of immune cells in the PCTS makes this model immunocompetent and therefore suitable for immunological investigations in the context of liver cancer [157,158] and pre-clinical testing of immunotherapeutics [146] and therapeutic vaccines, such as oncolytic measles vaccine viruses [150]. Another relevant feature of this organotypic model is that when PCTS are derived from human tissue, they preserve the patient-specific characteristics, including the original tumour histoarchitecture and proliferative capacity [151] as well as the individual responsiveness to therapy [159]. This similarity of the PCTS model to the tissue source is advantageous for predictive tests and the development of personalised therapies but also constitutes one of the main limitations. The reproducibility of the results is affected by the high variability among tumour phenotypes, subjects and susceptibility to treatment and as a consequence, large patient cohorts, a thorough experimental plan, and many technical replicates are necessary to build on consistent results. The essential requirement of multiple slices spatially distributed across the specimen is needed to overcome sampling error and compensate for the tumour heterogeneity. The inter-sample variability is relevant not only for human tissue but also for PCTS derived from mouse cancer models, as loco-regional changes in protein expression have been reported within a limited radius [160]. Unfortunately, the need to screen multiple slices and broaden the number of specimens, which depends on unpredictable factors, such as the availability and suitability of viable tissue, the challenging costs and time-consuming procedures impact the feasibility of these experiments.

Finally, compared to other models, PCTS survive in culture for a relatively short period, limiting their applicability, especially for drug screening. However, promising results have shown that employing innovative culture systems can extend the viability of liver slices [161], opening the possibility of using bioengineered bioreactors to advance the PCTS organotypic culture further.

### 3.5. Bioengineered Cancer Models

**Figure 1:**
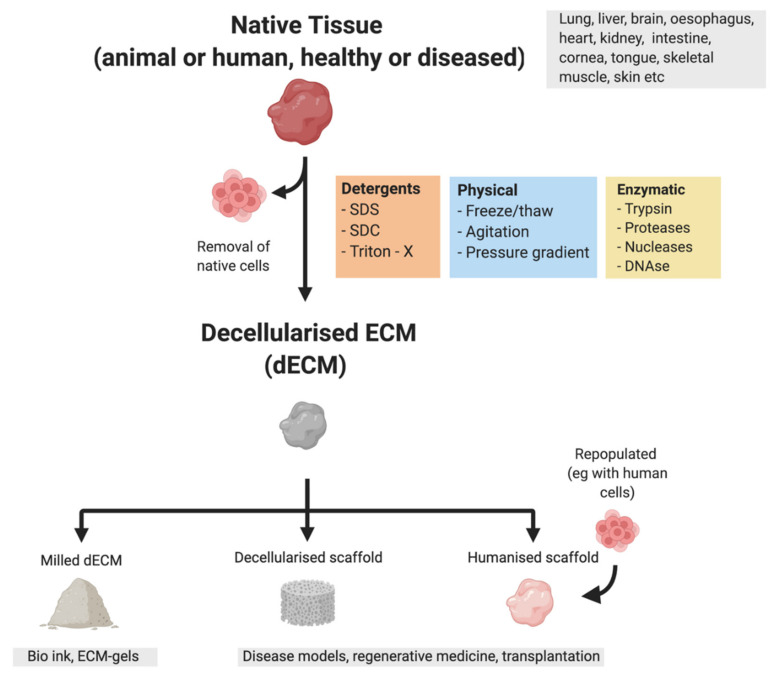
Most commonly used methods of decellularization and uses of decellularized ECM. Figure was created using BioRender (https://app.biorender.com, accessed 7 October 2021).

An emerging technology to study the effect of the ECM on cancer progression is bioengineered tissue models which can be made by combining tissue scaffolds and cells. These models utilise tissue engineering principles by seeding cells in a biocompatible scaffold along with a mixture of cytokines and growth factors required for the generation of the tissue [162]. Commonly used scaffolds currently include both animal-derived (e.g., Matrigel™) as well as synthetic hydrogels (e.g., PAG cryogel matrices) [162]. A promising way to generate human ECM-enriched bioscaffolds is through decellularization. Decellularization removes all cellular components from a tissue and allows for the isolation of patient-derived ECM [163] preserving the ECM structure along with all the ECM bound cytokines and growth factors (Figure 1). Decellularized ECM scaffolds can also be made into hydrogels applicable to 3D models [164]. Although decellularization is commonly used for bioengineering purposes [165,166,167], it also allows for the production of ECM-enriched tissue samples that can be analysed for their matrix composition using proteomics techniques. Using decellularized human colorectal cancer (CRC), CRLM and healthy liver and colon samples, Naba et al. were able to characterise the protein composition of these tissues. They found that the matrisome of CRLM is more similar to that of CRC than to healthy liver, highlighting the profound changes that the ECM undergoes during tumorigenesis [31]. Proteomics analysis of decellularized human cirrhotic liver showed a more abundant expression of several proteins including various collagens, LOXL1 and TGF-β-related proteins. Moreover, decellularized cirrhotic scaffolds promoted EMT when recellularized with a hepatoblastoma cell line and they also retained a higher amount of TGF-β compared to healthy scaffolds [168], emphasising how structural and component changes in the ECM can promote tumorigenesis.

Decellularization also enables the study of the sole effect of ECM components through the reduction of tissue into powder. Such an approach has been employed by Huleihel and colleagues who have extensively characterised macrophage phenotype in response to ECM powder stimuli. They stimulated both bone marrow-derived macrophages and the leukemic monocytic cell line THP-1 with ECM powder and found that ECM stimulus resulted in a distinct macrophage phenotype that differed from the classically (IFNγ + LPS) and alternatively (IL-4) activated phenotypes. Moreover, ECM stimulus drove macrophages towards an anti-inflammatory phenotype by downregulating inflammatory markers [169]. The use of decellularization to recapitulate features of the TME was demonstrated in an experiment using human CRC biopsies. The decellularization of these samples revealed that these matrices retain their biological properties and that following recellularization of CRC matrices with the colorectal adenocarcinoma cell line HT-29, there was an overexpression of IL-8, a chemokine essential for CRC growth [170]. A continuation of this study found that HT-29 cells cultured in CRLM scaffolds were more resistant to treatment with chemotherapy agents when used at a concentration determined in 2D cultures [171]. In another study, Mazza et al. demonstrated in a decellularized cirrhotic bioscaffold model, an enrichment of proteins affiliated with TGF-β/ECM related pathways and that the cirrhotic ECM contained higher endogenous amounts of TGF-β creating an environment that strongly favoured EMT compared to healthy bioscaffolds [168]. These studies highlight the importance of incorporating the ECM into cancer disease models and the difference its presence can make in determining the correct concentration of therapeutic compounds.

More widespread use of such techniques could guide clinicians in choosing the treatment with the highest predicted efficacy for each patient. Decellularized liver ECM has also been incorporated into an organ-on-chip model to study kidney cancer metastasis to the liver [172]. A biomimetic liver tumour-on-a-chip platform demonstrated better TME recapitulation by increasing hepatocyte viability and preserving their function when decellularized liver ECM was used in conjunction with GelMa [173]. This platform also showed a dose-dependent response to sorafenib and acetaminophen. Although, both rat liver ECMs used in the studies mentioned above demonstrate the potential these techniques hold for personalised medicine and for better modelling the liver TME during drug development.

While there is enormous potential in the use of decellularized scaffolds in bioengineered tumour models, there are several shortcomings affecting this technique. Naturally, there is patient-to-patient variation along with a lack of standardization for the protocols used to generate these scaffolds. There is also a difficulty in obtaining primary tissue and while there are already commercially available scaffolds, they are usually sourced from healthy individuals. Lastly, using these scaffolds makes it difficult to decipher the individual ECM components that can contribute to a pro-tumorigenic effect [174].

## 4. Conclusions

With rising liver cancer incidence worldwide, the need for new, efficacious treatments that are particularly effective in advanced cases is ever increasing. The success of approaches that combine immunotherapy with the targeting of the TME has highlighted the importance of considering the microenvironment during drug development. TME is an intercellular space that participates in various aspects of tumorigenesis including sustained proliferative signalling, angiogenesis, metastasis [65]. The TME is formed by both cellular and non-cellular components. The importance of a number of proteins of the ECM has been increasingly appreciated in driving tumorigenesis in various human carcinomas. The interplay between immune cells and ECM in the HCC TME is a key aspect that should be studied in respect to disease progression and therefore taken more into consideration in cancer models.

Because of the complex interplay between the tumour cells and the microenvironment, 2D models are not sufficient to reflect this complexity, therefore, the use of advanced models that incorporate the 3D tumour microenvironment are needed to obtain physiologically more insightful results. While the ECM’s function was for long regarded as simply providing structural support for tissues, it is becoming evident that its functions extended beyond that. The ECM sequesters growth factors, is a repository for cytokines, modulates the immune system [84] and is significantly remodelled during tumorigenesis. As 3D models mature, the interplay between the tumour and its microenvironment is becoming more appreciated. While commercially available matrices such as Matrigel are commonly used models such as organoids and spheroids, their composition can present with high batch-to-batch variation, and usually have non-human origins, making studies more difficult to compare to human disease mechanisms. A solution to this is offered by decellularization as it holds several advantages over currently used matrix substitutes. It allows for the isolation of patient-derived ECM while retaining core proteins, matrisome-associated proteins, cytokines, growth factors and its topology. This allows for the study of the specific environment in which the tumour developed making a more personalised approach possible. Nonetheless, this technique also suffers from limitations: it requires access to primary tissue, the heterogeneity is the same as between primary cells, which should be considered when comparing studies, and 3D models using decellularized ECM do not usually involve immune cells or they are not tumour specific. Currently, decellularized ECM studies are often carried out to assess changes in the behaviour of cancer cells in different matrices and to analyse the cancer matrix proteome, but not to assess the interaction between the ECM and the TIME. Although bioengineered models with decellularized ECM scaffolds are now more frequently used in cancer research to elucidate the role of ECM changes in cancer development and progression, their application to the study of HCC TME is rare. Proteomics analysis of ECM-enriched tumour samples to study the matrisome in HCC is also a far less used technique in comparison to other more studied solid cancers, such as CRC and CRLM. A deeper knowledge of specific matrix proteins present at different stages of the disease may help with the classification of HCC subtypes, the understanding of TME signalling pathways and the identification of pathways that could be targeted in new therapeutic strategies.

**Figure 2:**
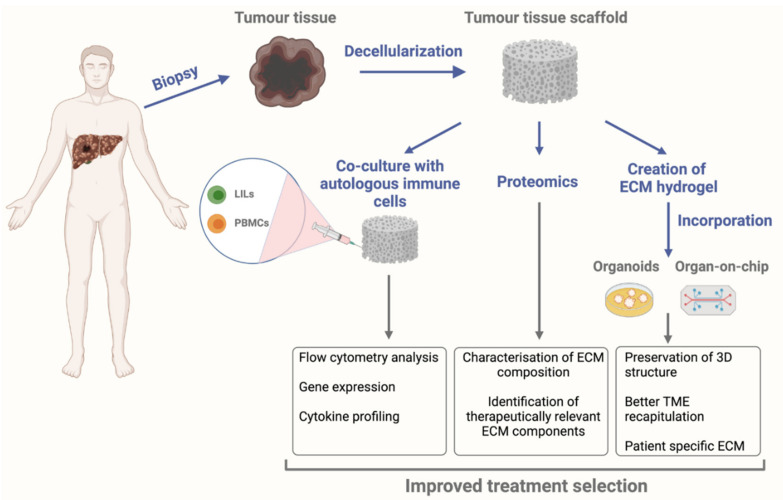
An example of a personalised bioengineered model that could be used to determine the best course of treatment for patients. In this model, we envisage that a tissue sample from the tumour site can be obtained using biopsy after which acellular scaffolds can be generated using decellularization. These tissue scaffolds can then have various applications including co-culture with autologous immune cells, analysis of composition with proteomics, or can be a source material for ECM hydrogels used in 3D disease models. LILs: liver infiltrating lymphocytes, PBMCs: peripheral blood mononuclear cells. The figure was created using BioRender (https://app.biorender.com, accessed 4 November 2021).

The use of decellularization for creating patient-specific matrices for bioengineered models could also open up new avenues in personalised medicine. A study performed in surgically resected CRC tissue that was decellularized and subsequently recellularized with CRC cell lines showed reduced sensitivity to 5-FU treatment and this reduce sensitivity was comparable to values in vivo [152]. Given the importance of the immune system in the TIME and its interaction with the remodelling ECM, disease-specific immune cells should be integrated into 3D models for the study of the TME. For example, a bioengineered model that consists of a patient’s ECM scaffold reseeded with autologous immune cells isolated from the tumour site or the surrounding tissue (Figure 2), could aid us in further exploring the mechanisms through which the diseased ECM affects anti-tumour immune responses and could help bring us a step closer in finding new therapeutic avenues for treating liver malignancies.

## Figures and Tables

**Table 1:** Main ECM remodelling processes in cancer in respect to normal ECM.

Increased ECM Deposition	ECM Degradation	Altered Post-Translational Modifications
↑Fibronectin, collagens I, III, IV	↑ECM degradation ↑Creation of bioactive molecules (matrikines) ↑Release of growth factors ↑Angiogenesis ↑ Metastasis	↑LOX activity ↑ECM stiffness ↑Mechanosignalling ↑Interstitial fluid pressure ↓ Impeded immune cell activity

↑ - increased; ↓ - decreased.

**Table 2:** Comparison of disease models.

Variation	2D Cell Lines	Spheroids	Organoids	Organ on Chip	Bioengineered Models	PCTS
Advantages	-Easy to use -Affordable -Highly reproducible -Long culture times	-Can be patient specific -Can be multicellular and be used in co-cultures -High reproducibility	-Patient specific -Various cell lineages -May contain tissue-specific ECM -Ability to co-culture	-Patient specific -Vascularisation -Ability for co-culture -Controlled environment	-Patient specific -Inclusion of native microenvironment -Ability for co-culture	-Preservation of in vivo microenvironment -Fully immunocompetent -Preservation of patient-specific characteristics
Disadvantages	-Lack of microenvironment -No cell heterogeneity -No patient specificity	-Poor structural organization -Forms a necrotic core -Limited oxygen and nutrients supply	-High inter patient variability -High cost -Lack of vascularization -Difficult to achieve maturation	-Low throughput -Difficulty with longevity -Technically challenging to use -Lack of technical robustness	-High inter patient variability -Lack of standardised protocols -Costly -Difficulty with obtaining tissue	-Short culture time -High inter patient variability -Difficulty with obtaining tissue

## References

[1] WHO GLOBOCAN Cancer Outlook. https://gco.iarc.fr/today/data/factsheets/cancers/11-Liver-fact-sheet.pdf.

[2] WHO (2016). Projections of Mortality and Causes of Death 2016 to 2060.

[3] Llovet J.M., Kelley R.K., Villanueva A., Singal A.G., Pikarsky E., Roayaie S., Lencioni R., Koike K., Zucman-Rossi J., Finn R.S. (2021). Hepatocellular carcinoma. Nat. Rev. Dis. Primers.

[4] Jemal A., Ward E.M., Johnson C.J., Cronin K.A., Ma J., Ryerson A.B., Mariotto A., Lake A.J., Wilson R., Sherman R.L. (2017). Annual Report to the Nation on the Status of Cancer, 1975–2014, Featuring Survival. J. Natl. Cancer Inst..

[5] Carloni V., Luong T.V., Rombouts K. (2014). Hepatic stellate cells and extracellular matrix in hepatocellular carcinoma: More complicated than ever. Liver Int..

[6] European Association for the Study of the Liver (2018). EASL Clinical Practice Guidelines: Management of hepatocellular carcinoma. J. Hepatol..

[7] SanGiovanni A., Prati G.M., Fasani P., Ronchi G., Romeo R., Manini M., Del Ninno E., Morabito A., Colombo M. (2006). The natural history of compensated cirrhosis due to hepatitis C virus: A 17-year cohort study of 214 patients. Hepatology.

[8] Craig A.J., Von Felden J., Garcia-Lezana T., Sarcognato S., Villanueva A. (2020). Tumour evolution in hepatocellular carcinoma. Nat. Rev. Gastroenterol. Hepatol..

[9] Tang T., Huang X., Zhang G., Hong Z., Bai X., Liang T. (2021). Advantages of targeting the tumor immune microenvironment over blocking immune checkpoint in cancer immunotherapy. Signal Transduct. Target. Ther..

[10] Villanueva A. (2019). Hepatocellular Carcinoma. N. Engl. J. Med..

[11] Llovet J.M., Villanueva A., Marrero J.A., Schwartz M., Meyer T., Galle P.R., Lencioni R., Greten T.F., Kudo M., Mandrekar S.J. (2021). Trial Design and Endpoints in Hepatocellular Carcinoma: AASLD Consensus Conference. Hepatology.

[12] El-Khoueiry A.B., Sangro B., Yau T., Crocenzi T.S., Kudo M., Hsu C., Kim T.-Y., Choo S.-P., Trojan J., Welling T.H. (2017). Nivolumab in patients with advanced hepatocellular carcinoma (CheckMate 040): An open-label, non-comparative, phase 1/2 dose escalation and expansion trial. Lancet.

[13] Zhu A.X., Finn R.S., Edeline J., Cattan S., Ogasawara S., Palmer D., Verslype C., Zagonel V., Fartoux L., Vogel A. (2018). Pembrolizumab in patients with advanced hepatocellular carcinoma previously treated with sorafenib (KEYNOTE-224): A non-randomised, open-label phase 2 trial. Lancet Oncol..

[14] Wainberg Z.A., Segal N.H., Jaeger D., Lee K.-H., Marshall J., Antonia S.J., Butler M., Sanborn R.E., Nemunaitis J.J., Carlson C.A. (2017). Safety and clinical activity of durvalumab monotherapy in patients with hepatocellular carcinoma (HCC). J. Clin. Oncol..

[15] Sangro B., Gomez-Martin C., de la Mata M., Iñarrairaegui M., Garralda E., Barrera P., Riezu-Boj J.-I., Larrea E., Alfaro C., Sarobe P. (2013). A clinical trial of CTLA-4 blockade with tremelimumab in patients with hepatocellular carcinoma and chronic hepatitis C. J. Hepatol..

[16] Yau T., Park J.W., Finn R.S., Cheng A.L., Mathurin P., Edeline J., Kudo M., Han K.H., Harding J.J., Merle P. (2019). CheckMate 459: A randomized, multi-center phase III study of nivolumab (NIVO) vs sorafenib (SOR) as first-line (1L) treatment in patients (pts) with advanced hepatocellular carcinoma (aHCC). Ann. Oncol..

[17] Finn R.S., Ryoo B.-Y., Merle P., Kudo M., Bouattour M., Lim H.-Y., Breder V.V., Edeline J., Chao Y., Ogasawara S. (2019). Results of KEYNOTE-240: Phase 3 study of pembrolizumab (Pembro) vs best supportive care (BSC) for second line therapy in advanced hepatocellular carcinoma (HCC). J. Clin. Oncol..

[18] Finn R.S., Qin S., Ikeda M., Galle P.R., Ducreux M., Kim T.-Y., Kudo M., Breder V., Merle P., Kaseb A.O. (2020). Atezolizumab plus Bevacizumab in Unresectable Hepatocellular Carcinoma. N. Engl. J. Med..

[19] Zhu A.X., Finn R.S., Ikeda M., Sung M.W., Baron A.D., Kudo M., Okusaka T., Kobayashi M., Kumada H., Kaneko S. (2020). A phase Ib study of lenvatinib (LEN) plus pembrolizumab (PEMBRO) in unresectable hepatocellular carcinoma (uHCC). J. Clin. Oncol..

[20] Kelley R.K., Cheng A.-L., Braiteh F.S., Park J.-W., Benzaghou F., Milwee S., Borgman A., El-Khoueiry A.B., Kayali Z.K., Zhu A.X. (2019). Phase 3 (COSMIC-312) study of cabozantinib (C) in combination with atezolizumab (A) versus sorafenib (S) in patients (pts) with advanced hepatocellular carcinoma (aHCC) who have not received previous systemic anticancer therapy. J. Clin. Oncol..

[21] Kelley R.K., Abou-Alfa G.K., Bendell J.C., Kim T.-Y., Borad M.J., Yong W.-P., Morse M., Kang Y.-K., Rebelatto M., Makowsky M. (2017). Phase I/II study of durvalumab and tremelimumab in patients with unresectable hepatocellular carcinoma (HCC): Phase I safety and efficacy analyses. J. Clin. Oncol..

[22] Yau T., Kang Y.-K., Kim T.-Y., El-Khoueiry A.B., Santoro A., Sangro B., Melero I., Kudo M., Hou M.-M., Matilla A. (2019). Nivolumab (NIVO) + ipilimumab (IPI) combination therapy in patients (pts) with advanced hepatocellular carcinoma (aHCC): Results from CheckMate. J. Clin. Oncol..

[23] Yin Z., Dong C., Jiang K., Xu Z., Li R., Guo K., Shao S., Wang L. (2019). Heterogeneity of cancer-associated fibroblasts and roles in the progression, prognosis, and therapy of hepatocellular carcinoma. J. Hematol. Oncol..

[24] Mouw J.K., Ou G., Weaver V.M. (2014). Extracellular matrix assembly: A multiscale deconstruction. Nat. Rev. Mol. Cell Biol..

[25] Ford A.J., Rajagopalan P. (2018). Extracellular matrix remodeling in 3D: Implications in tissue homeostasis and disease progression. Wiley Interdiscip. Rev. Nanomed. Nanobiotechnol..

[26] Charras G., Sahai E. (2014). Physical influences of the extracellular environment on cell migration. Nat. Rev. Mol. Cell Biol..

[27] Hastings J.F., Skhinas J.N., Fey D., Croucher D.R., Cox T.R. (2019). The extracellular matrix as a key regulator of intracellular signalling networks. Br. J. Pharmacol..

[28] Hay E.D. (1993). Extracellular matrix alters epithelial differentiation. Curr. Opin. Cell Biol..

[29] Berger C., Bjørlykke Y., Hahn L., Mühlemann M., Kress S., Walles H., Luxenhofer R., Ræder H., Metzger M., Zdzieblo D. (2020). Matrix decoded—A pancreatic extracellular matrix with organ specific cues guiding human iPSC differentiation. Biomaterials.

[30] Ueno T., Sata M., Tanikawa K., Oakazaki I., Ninomiya Y., Friedman S.L., Tanikawa K. (2003). Chapter 6—Cells Responsible for Extracellular Matrix Production in the Liver. Extracellular Matrix and the Liver.

[31] Naba A., Clauser K.R., Whittaker C.A., Carr S.A., Tanabe K.K., Hynes R.O. (2014). Extracellular matrix signatures of human primary metastatic colon cancers and their metastases to liver. BMC Cancer.

[32] Geervliet E., Bansal R. (2020). Matrix Metalloproteinases as Potential Biomarkers and Therapeutic Targets in Liver Diseases. Cells.

[33] Duarte S., Baber J., Fujii T., Coito A.J. (2015). Matrix metalloproteinases in liver injury, repair and fibrosis. Matrix Biol..

[34] Lu P., Takai K., Weaver V.M., Werb Z. (2011). Extracellular Matrix Degradation and Remodeling in Development and Disease. Cold Spring Harb. Perspect. Biol..

[35] Winkler J., Abisoye-Ogunniyan A., Metcalf K.J., Werb Z. (2020). Concepts of extracellular matrix remodelling in tumour progression and metastasis. Nat. Commun..

[36] Lu P., Weaver V.M., Werb Z. (2012). The extracellular matrix: A dynamic niche in cancer progression. J. Cell Biol..

[37] Ma H.-P., Chang H.-L., Bamodu O.A., Yadav V.K., Huang T.-Y., Wu A.T.H., Yeh C.-T., Tsai S.-H., Lee W.-H. (2019). Collagen 1A1 (COL1A1) Is a Reliable Biomarker and Putative Therapeutic Target for Hepatocellular Carcinogenesis and Metastasis. Cancers.

[38] Yang M.-C., Wang C.J., Liao P.-C., Yen C.-J., Shan Y.-S. (2014). Hepatic stellate cells secretes type I collagen to trigger epithelial mesenchymal transition of hepatoma cells. Am. J. Cancer Res..

[39] Levental K., Yu H., Kass L., Lakins J.N., Egeblad M., Erler J., Fong S.F., Csiszar K., Giaccia A., Weninger W. (2009). Matrix Crosslinking Forces Tumor Progression by Enhancing Integrin Signaling. Cell.

[40] Mustonen A.-M., Salvén A., Kärjä V., Rilla K., Matilainen J., Nieminen P. (2019). Hyaluronan histochemistry—A potential new tool to assess the progress of liver disease from simple steatosis to hepatocellular carcinoma. Glycobiology.

[41] Tanaka Y., Tateishi R., Koike K. (2018). Proteoglycans Are Attractive Biomarkers and Therapeutic Targets in Hepatocellular Carcinoma. Int. J. Mol. Sci..

[42] Dong Y., Xie X., Wang Z., Hu C., Zheng Q., Wang Y., Chen R., Xue T., Chen J., Gao D. (2014). Increasing matrix stiffness upregulates vascular endothelial growth factor expression in hepatocellular carcinoma cells mediated by integrin β1. Biochem. Biophys. Res. Commun..

[43] You Y., Zheng Q., Dong Y., Wang Y., Zhang L., Xue T., Xie X., Hu C., Wang Z., Chen R. (2015). Higher Matrix Stiffness Upregulates Osteopontin Expression in Hepatocellular Carcinoma Cells Mediated by Integrin β1/GSK3β/β-Catenin Signaling Pathway. PLoS ONE.

[44] Pan H.-W., Ou Y.-H., Peng S.-Y., Liu S.-H., Lai P.-L., Lee P.-H., Sheu J.-C., Chen C.-L., Hsu H.-C. (2003). Overexpression of osteopontin is associated with intrahepatic metastasis, early recurrence, and poorer prognosis of surgically resected hepatocellular carcinoma. Cancer.

[45] Yuzhalin A.E., Lim S.Y., Kutikhin A.G., Gordon-Weeks A.N. (2018). Dynamic matrisome: ECM remodeling factors licensing cancer progression and metastasis. Biochim. Biophys. Acta BBA Bioenerg..

[46] Ye M., Song Y., Pan S., Chu M., Wang Z.-W., Zhu X. (2020). Evolving roles of lysyl oxidase family in tumorigenesis and cancer therapy. Pharmacol. Ther..

[47] Wong C.C.-L., Tse A.P.-W., Huang Y.-P., Zhu Y.-T., Chiu D.K.-C., Lai R.K.-H., Au S.L.-K., Kai A.K.-L., Lee J.M.-F., Wei L.L. (2014). Lysyl oxidase-like 2 is critical to tumor microenvironment and metastatic niche formation in hepatocellular carcinoma. Hepatology.

[48] Cox T.R., Erler J.T. (2011). Remodeling and homeostasis of the extracellular matrix: Implications for fibrotic diseases and cancer. Dis. Model. Mech..

[49] Scheau C., Badarau I.A., Costache R., Caruntu C., Mihai G.L., Didilescu A.C., Constantin C., Neagu M. (2019). The Role of Matrix Metalloproteinases in the Epithelial-Mesenchymal Transition of Hepatocellular Carcinoma. Anal. Cell. Pathol..

[50] Wells J.M., Gaggar A., Blalock J.E. (2015). MMP generated matrikines. Matrix Biol..

[51] Brassart-Pasco S., Brézillon S., Brassart B., Ramont L., Oudart J.-B., Monboisse J.C. (2020). Tumor Microenvironment: Extracellular Matrix Alterations Influence Tumor Progression. Front. Oncol..

[52] Rivas M.J., Arii S., Furutani M., Harada T., Mizumoto M., Nishiyama H., Fujita J., Imamura M. (1998). Expression of human macrophage metalloelastase gene in hepatocellular carcinoma: Correlation with angiostatin generation and its clinical significance. Hepatology.

[53] Gorrin-Rivas M.J., Arii S., Mori A., Takeda Y., Mizumoto M., Furutani M., Imamura M. (2000). Implications of Human Macrophage Metalloelastase and Vascular Endothelial Growth Factor Gene Expression in Angiogenesis of Hepatocellular Carcinoma. Ann. Surg..

[54] Korpi J.T., Kervinen V., Mäklin H., Väänänen A., Lahtinen M., Läärä E., Ristimäki A., Thomas G., Ylipalosaari M., Åström P. (2008). Collagenase-2 (matrix metalloproteinase-8) plays a protective role in tongue cancer. Br. J. Cancer.

[55] Acuff H.B., Carter K.J., Fingleton B., Gorden D.L., Matrisian L.M. (2006). Matrix Metalloproteinase-9 from Bone Marrow–Derived Cells Contributes to Survival but not Growth of Tumor Cells in the Lung Microenvironment. Cancer Res..

[56] Zhao G., Cui J., Qin Q., Zhang J., Liu L., Deng S., Wu C., Yang M., Li S., Wang C. (2010). Mechanical stiffness of liver tissues in relation to integrin β1 expression may influence the development of hepatic cirrhosis and hepatocellular carcinoma. J. Surg. Oncol..

[57] Dong Y., Zheng Q., Wang Z., Lin X., You Y., Wu S., Wang Y., Hu C., Xie X., Chen J. (2019). Higher matrix stiffness as an independent initiator triggers epithelial-mesenchymal transition and facilitates HCC metastasis. J. Hematol. Oncol..

[58] Nguyen T.V., Sleiman M., Moriarty T., Herrick W.G., Peyton S.R. (2014). Sorafenib resistance and JNK signaling in carcinoma during extracellular matrix stiffening. Biomaterials.

[59] Schrader J., Gordon-Walker T.T., Aucott R.L., Van Deemter M., Quaas A., Walsh S., Benten D., Forbes S.J., Wells R.G., Iredale J.P. (2011). Matrix stiffness modulates proliferation, chemotherapeutic response, and dormancy in hepatocellular carcinoma cells. Hepatology.

[60] Kalluri R. (2016). The biology and function of fibroblasts in cancer. Nat. Rev. Cancer.

[61] Pelon F., Bourachot B., Kieffer Y., Magagna I., Mermet-Meillon F., Bonnet I., Costa A., Givel A.-M., Attieh Y., Barbazan J. (2020). Cancer-associated fibroblast heterogeneity in axillary lymph nodes drives metastases in breast cancer through complementary mechanisms. Nat. Commun..

[62] Chen X., Song E. (2019). Turning foes to friends: Targeting cancer-associated fibroblasts. Nat. Rev. Drug Discov..

[63] Deng Y., Cheng J., Fu B., Liu W., Chen G., Zhang Q., Yang Y. (2017). Hepatic carcinoma-associated fibroblasts enhance immune suppression by facilitating the generation of myeloid-derived suppressor cells. Oncogene.

[64] Li T., Yang Y., Hua X., Wang G., Liu W., Jia C., Tai Y., Zhang Q., Chen G. (2012). Hepatocellular carcinoma-associated fibroblasts trigger NK cell dysfunction via PGE2 and IDO. Cancer Lett..

[65] Barry A., Baldeosingh R., Lamm R., Patel K., Zhang K., Dominguez D.A., Kirton K.J., Shah A.P., Dang H. (2020). Hepatic Stellate Cells and Hepatocarcinogenesis. Front. Cell Dev. Biol..

[66] Thomson A.W., Knolle P.A. (2010). Antigen-presenting cell function in the tolerogenic liver environment. Nat. Rev. Immunol..

[67] Zhang J., Gu C., Song Q., Zhu M., Xu Y., Xiao M., Zheng W. (2020). Identifying cancer-associated fibroblasts as emerging targets for hepatocellular carcinoma. Cell Biosci..

[68] Refolo M.G., Messa C., Guerra V., Carr B.I., D’Alessandro R. (2020). Inflammatory Mechanisms of HCC Development. Cancers.

[69] Cannon J.G. (2000). Inflammatory Cytokines in Nonpathological States. Physiology.

[70] Lokau J., Schoeder V., Haybaeck J., Garbers C. (2019). Jak-Stat Signaling Induced by Interleukin-6 Family Cytokines in Hepatocellular Carcinoma. Cancers.

[71] Pickup M., Novitskiy S., Moses H.L. (2013). The roles of TGFbeta in the tumour microenvironment. Nat. Rev. Cancer.

[72] Chen J., Gingold J.A., Su X. (2019). Immunomodulatory TGF-β Signaling in Hepatocellular Carcinoma. Trends Mol. Med..

[73] Yamaguchi R., Yano H., Iemura A., Ogasawara S., Haramaki M., Kojiro M. (1998). Expression of vascular endothelial growth factor in human hepatocellular carcinoma. Hepatology.

[74] Poon R.T.-P., Ng I.O.-L., Lau C., Zhu L.-X., Yu W.-C., Lo C.-M., Fan S.-T., Wong J. (2001). Serum Vascular Endothelial Growth Factor Predicts Venous Invasion in Hepatocellular Carcinoma: A Prospective Study. Ann. Surg..

[75] Poon R.T.-P., Lau C., Yu W.-C., Fan S.-T., Wong J. (2004). High serum levels of vascular endothelial growth factor predict poor response to transarterial chemoembolization in hepatocellular carcinoma: A prospective study. Oncol. Rep..

[76] Zhao S., Wu D., Wu P., Wang Z., Huang J. (2015). Serum IL-10 Predicts Worse Outcome in Cancer Patients: A Meta-Analysis. PLoS ONE.

[77] Montanari N.R., Anugwom C.M., Boonstra A., Debes J.D. (2021). The Role of Cytokines in the Different Stages of Hepatocellular Carcinoma. Cancers.

[78] Binnewies M., Roberts E.W., Kersten K., Chan V., Fearon D.F., Merad M., Coussens L.M., Gabrilovich D.I., Ostrand-Rosenberg S., Hedrick C.C. (2018). Understanding the tumor immune microenvironment (TIME) for effective therapy. Nat. Med..

[79] Cassim S., Pouyssegur J. (2019). Tumor Microenvironment: A Metabolic Player that Shapes the Immune Response. Int. J. Mol. Sci..

[80] Yao W., He J.-C., Yang Y., Wang J.-M., Qian Y.-W., Yang T., Jian-Ming W. (2017). The Prognostic Value of Tumor-infiltrating Lymphocytes in Hepatocellular Carcinoma: A Systematic Review and Meta-analysis. Sci. Rep..

[81] Sia D., Jiao Y., Martinez-Quetglas I., Kuchuk O., Villacorta-Martin C., de Moura M.C., Putra J., Campreciós G., Bassaganyas L., Akers N. (2017). Identification of an Immune-specific Class of Hepatocellular Carcinoma, Based on Molecular Features. Gastroenterology.

[82] Gao X., Huang H., Wang Y., Pan C., Yin S., Zhou L., Zheng S. (2021). Tumor Immune Microenvironment Characterization in Hepatocellular Carcinoma Identifies Four Prognostic and Immunotherapeutically Relevant Subclasses. Front. Oncol..

[83] Bagaev A., Kotlov N., Nomie K., Svekolkin V., Gafurov A., Isaeva O., Osokin N., Kozlov I., Frenkel F., Gancharova O. (2021). Conserved pan-cancer microenvironment subtypes predict response to immunotherapy. Cancer Cell.

[84] McQuitty C.E., Williams R., Chokshi S., Urbani L. (2020). Immunomodulatory Role of the Extracellular Matrix Within the Liver Disease Microenvironment. Front. Immunol..

[85] Hinz B. (2015). The extracellular matrix and transforming growth factor-β1: Tale of a strained relationship. Matrix Biol..

[86] Fabregat I., Caballero-Díaz D. (2018). Transforming Growth Factor-β-Induced Cell Plasticity in Liver Fibrosis and Hepatocarcinogenesis. Front. Oncol..

[87] Thomas D.A., Massagué J. (2005). TGF-β directly targets cytotoxic T cell functions during tumor evasion of immune surveillance. Cancer Cell.

[88] Zheng S.G., Gray J.D., Ohtsuka K., Yamagiwa S., Horwitz D.A. (2002). Generation ex vivo of TGF-β-producing regulatory T cells from CD4+CD25− precursors. J. Immunol..

[89] Chen W., Jin W., Hardegen N.J., Lei K.-J., Li J., Marinos N.J., McGrady G., Wahl S.M. (2003). Conversion of Peripheral CD4+CD25− Naive T Cells to CD4+CD25+ Regulatory T Cells by TGF-β Induction of Transcription Factor Foxp3. J. Exp. Med..

[90] Salmon H., Franciszkiewicz K., Damotte D., Dieu-Nosjean M.-C., Validire P., Trautmann A., Mami-Chouaib F., Donnadieu E. (2012). Matrix architecture defines the preferential localization and migration of T cells into the stroma of human lung tumors. J. Clin. Investig..

[91] Nicolas-Boluda A., Donnadieu E. (2019). Obstacles to T cell migration in the tumor microenvironment. Comp. Immunol. Microbiol. Infect. Dis..

[92] Murdamoothoo D., Sun Z., Yilmaz A., Riegel G., Abou-Faycal C., Deligne C., Velazquez-Quesada I., Erne W., Nascimento M., Mörgelin M. (2021). Tenascin-C immobilizes infiltrating T lymphocytes through CXCL12 promoting breast cancer progression. EMBO Mol. Med..

[93] Elahi-Gedwillo K.Y., Carlson M., Zettervall J., Provenzano P.P. (2019). Antifibrotic Therapy Disrupts Stromal Barriers and Modulates the Immune Landscape in Pancreatic Ductal Adenocarcinoma. Cancer Res..

[94] Hartmann N., Giese N.A., Giese T., Poschke I., Offringa R., Werner J., Ryschich E. (2014). Prevailing Role of Contact Guidance in Intrastromal T-cell Trapping in Human Pancreatic Cancer. Clin. Cancer Res..

[95] Kuczek D.E., Larsen A.M.H., Thorseth M.-L., Carretta M., Kalvisa A., Siersbæk M.S., Simões A.M.C., Roslind A., Engelholm L.H., Noessner E. (2019). Collagen density regulates the activity of tumor-infiltrating T cells. J. Immunother. Cancer.

[96] Peng D.H., Rodriguez B.L., Diao L., Chen L., Wang J., Byers L.A., Wei Y., Chapman H.A., Yamauchi M., Behrens C. (2020). Collagen promotes anti-PD-1/PD-L1 resistance in cancer through LAIR1-dependent CD8+ T cell exhaustion. Nat. Commun..

[97] Klement J., Poschel D., Lu C., Merting A., Yang D., Redd P., Liu K. (2021). Osteopontin Blockade Immunotherapy Increases Cytotoxic T Lymphocyte Lytic Activity and Suppresses Colon Tumor Progression. Cancers.

[98] Noda Y., Kishino M., Sato S., Hirose K., Sakai M., Fukuda Y., Murakami S., Toyosawa S. (2016). Galectin-1 expression is associated with tumour immunity and prognosis in gingival squamous cell carcinoma. J. Clin. Pathol..

[99] Nambiar D.K., Aguilera T., Cao H., Kwok S., Kong C., Bloomstein J., Wang Z., Rangan V.S., Jiang D., Von Eyben R. (2019). Galectin-1–driven T cell exclusion in the tumor endothelium promotes immunotherapy resistance. J. Clin. Investig..

[100] Daley D., Mani V.R., Mohan N., Akkad N., Ochi A., Heindel D.W., Lee K.B., Zambirinis C.P., Pandian G.S.B., Savadkar S. (2017). Dectin 1 activation on macrophages by galectin 9 promotes pancreatic carcinoma and peritumoral immune tolerance. Nat. Med..

[101] Mariathasan S., Turley S.J., Nickles D., Castiglioni A., Yuen K., Wang Y., Kadel E.E., Koeppen H., Astarita J.L., Cubas R. (2018). TGFβ attenuates tumour response to PD-L1 blockade by contributing to exclusion of T cells. Nature.

[102] Lee W.S., Yang H., Chon H.J., Kim C. (2020). Combination of anti-angiogenic therapy and immune checkpoint blockade normalizes vascular-immune crosstalk to potentiate cancer immunity. Exp. Mol. Med..

[103] Dirkx A.E., oude Egbrink M.G., Kuijpers M.J., van der Niet S.T., Heijnen V.V., Bouma-ter Steege J.C., Wagstaff J., Griffioen A.W. (2003). Tumor Angiogenesis Modulates Leukocyte-Vessel Wall Interactions in vivo by Reducing Endothelial Adhesion Molecule Expression. Cancer Res..

[104] Tromp S.C., Egbrink M.G.A.O., Dings R., Van Velzen S., Slaaf D.W., Hillen H.F.P., Tangelder G.J., Reneman R.S., Griffioen A.W. (2000). Tumor angiogenesis factors reduce leukocyte adhesion in vivo. Int. Immunol..

[105] Lanitis E., Irving M., Coukos G. (2015). Targeting the tumor vasculature to enhance T cell activity. Curr. Opin. Immunol..

[106] Dirkx A.E.M., Egbrink M.G.A.O., Castermans K., van der Schaft D.W.J., Thijssen V.L.J.L., Dings R.P.M., Kwee L., Mayo K.H., Wagstaff J., Steege J.C.A.B. (2006). Anti-angiogenesis therapy can overcome endothelial cell anergy and promote leukocyte-endothelium interactions and infiltration in tumors. FASEB J..

[107] McKeown S.R. (2014). Defining normoxia, physoxia and hypoxia in tumours—implications for treatment response. Br. J. Radiol..

[108] Méndez-Blanco C., Fondevila F., Palomo A.G., González-Gallego J., Mauriz J.L. (2018). Sorafenib resistance in hepatocarcinoma: Role of hypoxia-inducible factors. Exp. Mol. Med..

[109] Arrowsmith J., Miller P. (2013). Phase II and Phase III attrition rates 2011–2012. Nat. Rev. Drug Discov..

[110] Costa E.C., Moreira A.F., Diogo D.M.D.M., Gaspar V., Carvalho M.P., Correia I.J. (2016). 3D tumor spheroids: An overview on the tools and techniques used for their analysis. Biotechnol. Adv..

[111] Porter R.J., Murray G.I., McLean M.H. (2020). Current concepts in tumour-derived organoids. Br. J. Cancer.

[112] Lv D., Hu Z., Lu L., Lu H., Xu X. (2017). Three-dimensional cell culture: A powerful tool in tumor research and drug discovery (Review). Oncol. Lett..

[113] Langhans S.A. (2018). Three-Dimensional in Vitro Cell Culture Models in Drug Discovery and Drug Repositioning. Front. Pharmacol..

[114] Petersen O.W., Rønnov-Jessen L., Howlett A.R., Bissell M.J., nbsp (1992). Interaction with basement membrane serves to rapidly distinguish growth and differentiation pattern of normal and malignant human breast epithelial cells. Proc. Natl. Acad. Sci. USA.

[115] Bissell M.J., Radisky D. (2001). Putting tumours in context. Nat. Rev. Cancer.

[116] Fischbach C., Kong H.J., Hsiong S.X., Evangelista M.B., Yuen W., Mooney D.J., nbsp (2009). Cancer cell angiogenic capability is regulated by 3D culture and integrin engagement. Proc. Natl. Acad. Sci. USA.

[117] Longati P., Jia X., Eimer J., Wagman A., Witt M.-R., Rehnmark S., Verbeke C., Toftgård R., Löhr M., Heuchel R.L. (2013). 3D pancreatic carcinoma spheroids induce a matrix-rich, chemoresistant phenotype offering a better model for drug testing. BMC Cancer.

[118] Lagies S., Schlimpert M., Neumann S., Wäldin A., Kammerer B., Borner C., Peintner L. (2020). Cells grown in three-dimensional spheroids mirror in vivo metabolic response of epithelial cells. Commun. Biol..

[119] Melissaridou S., Wiechec E., Magan M., Jain M.V., Chung M.K., Farnebo L., Roberg K. (2019). The effect of 2D and 3D cell cultures on treatment response, EMT profile and stem cell features in head and neck cancer. Cancer Cell Int..

[120] Riedl A., Schlederer M., Pudelko K., Stadler M., Walter S., Unterleuthner D., Unger C., Kramer N., Hengstschläger M., Kenner L. (2017). Comparison of cancer cells in 2D vs 3D culture reveals differences in AKT–mTOR–S6K signaling and drug responses. J. Cell Sci..

[121] Lancaster M.A., Knoblich J.A. (2014). Organogenesis in a dish: Modeling development and disease using organoid technologies. Sci..

[122] Huch M., Gehart H., van Boxtel R., Hamer K., Blokzijl F., Verstegen M.M., Ellis E., Van Wenum M., Fuchs S.A., de Ligt J. (2015). Long-Term Culture of Genome-Stable Bipotent Stem Cells from Adult Human Liver. Cell.

[123] Broutier L., Mastrogiovanni G., Verstegen M.M.A., Francies H.E., Gavarró L.M., Bradshaw C.R., Allen G.E., Arnes-Benito R., Sidorova O., Gaspersz M.P. (2017). Human primary liver cancer–derived organoid cultures for disease modeling and drug screening. Nat. Med..

[124] Finnberg N.K., Gokare P., Lev A., Grivennikov S.I., MacFarlane A.W., Campbell K.S., Winters R.M., Kaputa K., Farma J.M., Abbas E.-S.A. (2017). Application of 3D tumoroid systems to define immune and cytotoxic therapeutic responses based on tumoroid and tissue slice culture molecular signatures. Oncotarget.

[125] Nozaki K., Mochizuki W., Matsumoto Y., Matsumoto T., Fukuda M., Mizutani T., Watanabe M., Nakamura T. (2016). Co-culture with intestinal epithelial organoids allows efficient expansion and motility analysis of intraepithelial lymphocytes. J. Gastroenterol..

[126] Pasch C.A., Favreau P.F., Yueh A.E., Babiarz C.P., Gillette A.A., Sharick J.T., Karim M.R., Nickel K.P., DeZeeuw A.K., Sprackling C.M. (2019). Patient-Derived Cancer Organoid Cultures to Predict Sensitivity to Chemotherapy and Radiation. Clin. Cancer Res..

[127] Ooft S.N., Weeber F., Dijkstra K.K., McLean C.M., Kaing S., Van Werkhoven E., Schipper L., Hoes L., Vis D.J., Van De Haar J. (2019). Patient-derived organoids can predict response to chemotherapy in metastatic colorectal cancer patients. Sci. Transl. Med..

[128] Bruun J., Kryeziu K., Eide P.W., Moosavi S.H., Eilertsen I.A., Langerud J., Røsok B.I., Totland M.Z., Brunsell T.H., Pellinen T. (2020). Patient-Derived Organoids from Multiple Colorectal Cancer Liver Metastases Reveal Moderate Intra-patient Pharmacotranscriptomic Heterogeneity. Clin. Cancer Res..

[129] Liu J., Li P., Wang L., Li M., Ge Z., Noordam L., Lieshout R., Verstegen M.M., Ma B., Su J. (2021). Cancer-Associated Fibroblasts Provide a Stromal Niche for Liver Cancer Organoids That Confers Trophic Effects and Therapy Resistance. Cell. Mol. Gastroenterol. Hepatol..

[130] Białkowska K., Komorowski P., Bryszewska M., Miłowska K. (2020). Spheroids as a Type of Three-Dimensional Cell Cultures—Examples of Methods of Preparation and the Most Important Application. Int. J. Mol. Sci..

[131] Amann T., Bataille F., Spruss T., Mühlbauer M., Gäbele E., Schölmerich J., Kiefer P., Bosserhoff A.-K., Hellerbrand C. (2009). Activated hepatic stellate cells promote tumorigenicity of hepatocellular carcinoma. Cancer Sci..

[132] Jung H.R., Kang H.M., Hong-Ryul J., Kim D.-S., Noh K.H., Kim E.-S., Lee H.-J., Chung K.-S., Cho H.-S., Kim N.-S. (2017). Cell Spheroids with Enhanced Aggressiveness to Mimic Human Liver Cancer In Vitro and In Vivo. Sci. Rep..

[133] Song Y., Kim S.-H., Kim K.M., Choi E.K., Kim J., Seo H.R. (2016). Activated hepatic stellate cells play pivotal roles in hepatocellular carcinoma cell chemoresistance and migration in multicellular tumor spheroids. Sci. Rep..

[134] Song Y., Kim J.-S., Kim S.-H., Park Y.K., Yu E., Kim K.-H., Seo E.-J., Oh H.-B., Lee H.C., Kim K.M. (2018). Patient-derived multicellular tumor spheroids towards optimized treatment for patients with hepatocellular carcinoma. J. Exp. Clin. Cancer Res..

[135] Ingber D.E. (2018). Developmentally inspired human ‘organs on chips’. Development.

[136] Beckwitt C., Clark A., Wheeler S., Taylor D.L., Stolz D.B., Griffith L., Wells A. (2018). Liver ‘organ on a chip’. Exp. Cell Res..

[137] Benam K.H., Villenave R., Lucchesi C., Varone A., Hubeau C., Lee H.-H., Alves S.E., Salmon M., Ferrante T.C., Weaver J.C. (2016). Small airway-on-a-chip enables analysis of human lung inflammation and drug responses in vitro. Nat. Methods.

[138] Stucki A.O., Stucki J.D., Hall S.R.R., Felder M., Mermoud Y., Schmid R.A., Geiser T., Guenat O.T. (2015). A lung-on-a-chip array with an integrated bio-inspired respiration mechanism. Lab Chip.

[139] Sun W., Luo Z., Lee J., Kim H.-J., Lee K., Tebon P., Feng Y., Dokmeci M.R., Sengupta S., Khademhosseini A. (2019). Organ-on-a-Chip for Cancer and Immune Organs Modeling. Adv. Healthc. Mater..

[140] Sontheimer-Phelps A., Hassell B.A., Ingber D.E. (2019). Modelling cancer in microfluidic human organs-on-chips. Nat. Rev. Cancer.

[141] Ayuso J.M., Rehman S., Virumbrales-Munoz M., McMinn P.H., Geiger P., Fitzgerald C., Heaster T., Skala M.C., Beebe D.J. (2021). Microfluidic tumor-on-a-chip model to evaluate the role of tumor environmental stress on NK cell exhaustion. Sci. Adv..

[142] Aref A.R., Campisi M., Ivanova E., Portell A., Larios D., Piel B.P., Mathur N., Zhou C., Coakley R.V., Bartels A. (2018). 3D microfluidic ex vivo culture of organotypic tumor spheroids to model immune checkpoint blockade. Lab Chip.

[143] Businaro L., De Ninno A., Schiavoni G., Lucarini V., Ciasca G., Gerardino A., Belardelli F., Gabriele L., Mattei F. (2013). Cross talk between cancer and immune cells: Exploring complex dynamics in a microfluidic environment. Lab Chip.

[144] Misra S., Moro C.F., Del Chiaro M., Pouso S., Sebestyén A., Löhr M., Björnstedt M., Verbeke C.S. (2019). Ex vivo organotypic culture system of precision-cut slices of human pancreatic ductal adenocarcinoma. Sci. Rep..

[145] Roelants C., Pillet C., Franquet Q., Sarrazin C., Peilleron N., Giacosa S., Guyon L., Fontanell A., Fiard G., Long J.-A. (2020). Ex-Vivo Treatment of Tumor Tissue Slices as a Predictive Preclinical Method to Evaluate Targeted Therapies for Patients with Renal Carcinoma. Cancers.

[146] Sivakumar R., Chan M., Shin J.S., Nishida-Aoki N., Kenerson H.L., Elemento O., Beltran H., Yeung R., Gujral T.S. (2019). Organotypic tumor slice cultures provide a versatile platform for immuno-oncology and drug discovery. OncoImmunology.

[147] Kishan A.T.N., Nicole S.V., Humberto S., van Deurzen Carolien H.M., den Bakker Michael A., Jan H.J.H., Roland K., Vreeswijk Maaike P.G., Agnes J., van Gent D.C. (2016). Tumor slice culture system to assess drug response of primary breast cancer. BMC Cancer.

[148] Kenerson H.L., Sullivan K.M., Seo Y.D., Stadeli K.M., Ussakli C., Yan X., Lausted C., Pillarisetty V.G., Park J.O., Riehle K.J. (2020). Tumor slice culture as a biologic surrogate of human cancer. Ann. Transl. Med..

[149] Roife D., Dai B., Kang Y., Perez M.V.R., Pratt M., Li X., Fleming J.B. (2016). Ex Vivo Testing of Patient-Derived Xenografts Mirrors the Clinical Outcome of Patients with Pancreatic Ductal Adenocarcinoma. Clin. Cancer Res..

[150] Zimmermann M., Armeanu S., Smirnow I., Kupka S., Wagner S., Wehrmann M., Rots M.G., Groothuis G.M.M., Weiss T.S., Königsrainer A. (2009). Human precision-cut liver tumor slices as a tumor patient-individual predictive test system for oncolytic measles vaccine viruses. Int. J. Oncol..

[151] Doornebal E., Harris N., Cooksley H., Pizanias M., Miquel R., Zen Y., Zamalloa A., Preziosi M., Heaton N., Prachalias A. (2020). Development of personalised human immunocompetent ex vivo models of primary and secondary liver cancers using precision cut tissue slice technology. J. Hepatol..

[152] Cassim S., Raymond V.-A., Lacoste B., Lapierre P., Bilodeau M. (2018). Metabolite profiling identifies a signature of tumorigenicity in hepatocellular carcinoma. Oncotarget.

[153] Palma E., Doornebal E.J., Chokshi S. (2019). Precision-cut liver slices: A versatile tool to advance liver research. Hepatol. Int..

[154] Graaf I.A.M.D., Olinga P., De Jager M.H., Merema M.T., de Kanter R., Van De Kerkhof E.G., Groothuis G.M.M. (2010). Preparation and incubation of precision-cut liver and intestinal slices for application in drug metabolism and toxicity studies. Nat. Protoc..

[155] Palma E., Riva A., Moreno C., Odena G., Mudan S., Manyakin N., Miquel R., Degré D., Trepo E., Sancho-Bru P. (2020). Perturbations in Mitochondrial Dynamics Are Closely Involved in the Progression of Alcoholic Liver Disease. Alcohol. Clin. Exp. Res..

[156] Sadasivan S.K., Nethra S., Khan K.M., Vasamsetti B., Kumar N.R., Haridas V., Reddy M.B., Somesh B.P., Oommen A., Rao R.P. (2015). Developing an in vitro screening assay platform for evaluation of antifibrotic drugs using precision-cut liver slices. Fibrogenes. Tissue Repair.

[157] Wu X., Roberto J.B., Knupp A., Kenerson H.L., Truong C.D., Yuen S.Y., Brempelis K.J., Tuefferd M., Chen A., Horton H. (2018). Precision-cut human liver slice cultures as an immunological platform. J. Immunol. Methods.

[158] Stärkel P., Schnabl B., Leclercq S., Komuta M., Bataller R., Argemi J., Palma E., Chokshi S., Hellerbrand C., Maccioni L. (2019). Deficient IL-6/Stat3 Signaling, High TLR7, and Type I Interferons in Early Human Alcoholic Liver Disease: A Triad for Liver Damage and Fibrosis. Hepatol. Commun..

[159] Voabil P., de Bruijn M., Roelofsen L.M., Hendriks S.H., Brokamp S., Braber M.V.D., Broeks A., Sanders J., Herzig P., Zippelius A. (2021). An ex vivo tumor fragment platform to dissect response to PD-1 blockade in cancer. Nat. Med..

[160] Davies E.J., Dong M., Gutekunst M., Närhi K., Van Zoggel H.J.A.A., Blom S., Nagaraj A., Metsalu T., Oswald E., Erkens-Schulze S. (2015). Capturing complex tumour biology in vitro: Histological and molecular characterisation of precision cut slices. Sci. Rep..

[161] Paish H.L., Reed L.H., Brown H., Bryan M.C., Govaere O., Leslie J., Barksby B.S., Macia M.G., Watson A., Xu X. (2019). A Bioreactor Technology for Modeling Fibrosis in Human and Rodent Precision-Cut Liver Slices. Hepatology.

[162] Moysidou C.-M., Barberio C., Owens R.M. (2021). Advances in Engineering Human Tissue Models. Front. Bioeng. Biotechnol..

[163] Crapo P.M., Gilbert T.W., Badylak S.F. (2011). An overview of tissue and whole organ decellularization processes. Biomaterials.

[164] Giobbe G.G., Crowley C., Luni C., Campinoti S., Khedr M., Kretzschmar K., De Santis M.M., Zambaiti E., Michielin F., Meran L. (2019). Extracellular matrix hydrogel derived from decellularized tissues enables endodermal organoid culture. Nat. Commun..

[165] Ott H.C., Matthiesen T.S., Goh S.-K., Black L.D., Kren S., Netoff T.I., Taylor D.A. (2008). Perfusion-decellularized matrix: Using nature’s platform to engineer a bioartificial heart. Nat. Med..

[166] Uygun B., Soto-Gutierrez A., Yagi H., Izamis M.-L., Guzzardi M., Shulman C., Milwid J., Kobayashi N., Tilles A., Berthiaume F. (2010). Organ reengineering through development of a transplantable recellularized liver graft using decellularized liver matrix. Nat. Med..

[167] Petersen T.H., Calle E.A., Zhao L., Lee E.J., Gui L., Raredon M.B., Gavrilov K., Yi T., Zhuang Z.W., Breuer C. (2010). Tissue-Engineered Lungs for in Vivo Implantation. Science.

[168] Mazza G., Telese A., Al-Akkad W., Frenguelli L., Levi A., Marrali M., Longato L., Thanapirom K., Vilia M.G., Lombardi B. (2019). Cirrhotic Human Liver Extracellular Matrix 3D Scaffolds Promote Smad-Dependent TGF-β1 Epithelial Mesenchymal Transition. Cells.

[169] Huleihel L., Dziki J.L., Bartolacci J.G., Rausch T., Scarritt M.E., Cramer M.C., Vorobyov T., LoPresti S.T., Swineheart I.T., White L. (2017). Macrophage phenotype in response to ECM bioscaffolds. Semin. Immunol..

[170] Piccoli M., D’Angelo E., Crotti S., Sensi F., Urbani L., Maghin E., Burns A., De Coppi P., Fassan M., Rugge M. (2018). Decellularized colorectal cancer matrix as bioactive microenvironment for in vitro 3D cancer research. J. Cell. Physiol..

[171] D’Angelo E., Natarajan D., Sensi F., Ajayi O., Fassan M., Mammano E., Pilati P., Pavan P., Bresolin S., Preziosi M. (2020). Patient-Derived Scaffolds of Colorectal Cancer Metastases as an Organotypic 3D Model of the Liver Metastatic Microenvironment. Cancers.

[172] Wang Y., Wu D., Wu G., Wu J., Lu S., Lo J., He Y., Zhao C., Zhao X., Zhang H. (2020). Metastasis-on-a-chip mimicking the progression of kidney cancer in the liver for predicting treatment efficacy. Theranostics.

[173] Lu S., Cuzzucoli F., Jiang J., Liang L.-G., Wang Y., Kong M., Zhao X., Cui W., Li J., Wang S. (2018). Development of a biomimetic liver tumor-on-a-chip model based on decellularized liver matrix for toxicity testing. Lab Chip.

[174] Micek H.M., Visetsouk M.R., Masters K.S., Kreeger P.K. (2020). Engineering the Extracellular Matrix to Model the Evolving Tumor Microenvironment. iScience.

